# Socio-economic and insurance factors in preventive care: A two-stage weighted structural analysis

**DOI:** 10.1016/j.dialog.2025.100243

**Published:** 2025-10-06

**Authors:** Florent Nkouaga

**Affiliations:** National Association of Insurance Commissioners (NAIC), Kansas City, MO, USA

**Keywords:** Health insurance, Preventive health, Psychological price, Unexpected bills, Primary care, Financial knowledge

## Abstract

This study investigates the key determinants influencing the utilization of preventive health services among insured individuals in the United States following the implementation of the Affordable Care Act (ACA). Leveraging nationally representative data from the 2024 Financial Inclusion Survey, the research employs weighted logistic regression and Structural Equation Modeling (SEM) to analyze how perceived insurance coverage quality, access to a primary care physician, financial knowledge, and experiences with healthcare costs are associated with the uptake of no-cost preventive care. The findings reveal that individuals who report higher satisfaction with their insurance coverage, maintain ongoing relationships with primary care providers, and demonstrate greater financial literacy are more likely to engage in preventive health behaviors. Notably, while cost-related barriers are initially associated with increased utilization, their direct influence diminishes when financial knowledge is accounted for, highlighting the mitigating role of financial literacy. The SEM approach further clarifies the complex interplay between latent constructs such as perceived coverage quality and cost experiences, showing that these factors interact with each other and with other determinants to shape preventive care decisions. Importantly, once these structural and informational dimensions are considered, traditional demographic variables — such as race and gender — lose statistical significance, underscoring the greater impact of systemic and educational factors. These results suggest that policies aimed solely at eliminating financial barriers may be insufficient to maximize preventive care participation. Instead, fostering trust in insurance coverage, strengthening primary care relationships, and enhancing financial literacy are critical strategies for increasing preventive service utilization. By addressing the informational, relational, and economic dimensions of healthcare access, policymakers and stakeholders can more effectively promote preventive health engagement and advance population health outcomes.

## 1 Introduction

Preventive health services — ranging from immunizations and screenings to counseling and early interventions — are widely recognized as essential for reducing morbidity, mortality, and long-term healthcare costs in the United States [1], [2], [3], [4], [5]. Historically, the uptake of preventive care has lagged behind public health goals, with persistent disparities across socioeconomic and demographic groups [6], [7], [8], [9]. In response, the Affordable Care Act (ACA), enacted in 2010, marked a pivotal policy shift by mandating that most private insurance plans, Medicare, and Medicaid expansion programs cover over 100 preventive services without cost-sharing [1], [2], [10], [11], [12], [13], [14]. This legislative overhaul aimed to eliminate financial barriers, thereby increasing access and utilization of preventive care for millions—including 150 million with commercial insurance, 61 million on Medicare, and 20 million Medicaid expansion enrollees [1], [11], [15].

The ACA’s preventive care mandate has yielded measurable improvements: studies document increased rates of cancer screenings, immunizations, and chronic disease management, particularly among women, low-income groups, and those served by community health centers [11], [13], [16], [17], [18]. The public health impact is substantial, as preventive services have the potential to avert nine of the ten leading causes of death and save over 100,000 lives annually [11], [19]. However, despite these advances, utilization rates remain suboptimal. Only about half of recommended preventive services are used, even when available at no cost [17], [20], [21], [22]. This persistent underuse suggests that factors beyond financial barriers — such as health insurance literacy, perceived coverage quality, access to primary care, and experiences with healthcare costs — play critical roles in shaping preventive health behaviors [23], [24], [25], [26], [27].

Moreover, the landscape of preventive care remains dynamic and challenged by ongoing policy debates, legal disputes over the ACA’s provisions, and implementation gaps exacerbated by the COVID-19 pandemic [28], [29], [30], [31], [32], [33], [34], [35]. Disparities in access and utilization persist among racial and ethnic minorities, individuals of lower socioeconomic status, and those with limited English proficiency, who are less likely to receive recommended preventive care [7], [8], [9]. These gaps are further complicated by systemic factors, such as provider shortages, transportation barriers, and unbalanced financial incentives that often prioritize treatment over prevention [10], [36].

Addressing these challenges requires a multifaceted, policy-focused approach. Researchers and policymakers advocate for strategies including value-based payment models, enhanced health literacy initiatives, culturally competent interventions, and improved data collection to monitor preventive service use across diverse populations [37], [38], [39]. As the U.S. healthcare system continues to evolve, it is imperative to understand not only the impact of removing financial barriers, but also the broader determinants that drive or inhibit preventive health service utilization.

This research is centered on identifying and analyzing the key determinants that influence the utilization of preventive health services among insured individuals in the United States, with a particular emphasis on perceived coverage quality, health access cost experience, access to a primary care physician, and financial knowledge. Leveraging nationally representative data from the 2024 Financial Inclusion Survey, the study offers a comprehensive and contemporary analysis of how these factors interact to shape the uptake of no-cost preventive services in the post-ACA landscape. By focusing on current utilization patterns within the U.S. context, the research provides timely policy-relevant insights into the drivers of preventive care engagement, setting the stage for future efforts to enhance the effectiveness and reach of preventive health initiatives.

*Hypotheses:*

1 *Perceived coverage quality* is associated with preventive health service utilization and may have a complex relationship with usage, including the possibility that higher perceived generosity of coverage could be linked to reduced urgency to seek preventive care.
2 *Health access cost experience* is expected to be related to the use of preventive care, but its direct association may be diminished or become non-significant when financial knowledge is considered.
3 *Access to a primary care physician* is strongly and positively associated with the utilization of preventive health services, highlighting the importance of patient-provider continuity.
4 *Financial knowledge* is a robust positive correlate of preventive care use, both through direct association with utilization and by being linked to lower perceived cost barriers.

By clarifying these relationships, the study seeks to inform policy strategies that move beyond eliminating financial barriers alone, emphasizing the importance of informational, relational, and experiential factors in advancing the ACA’s public health objectives.

## 2 Theory

This study examines the various factors influencing the adoption of preventive health services among insured individuals in the United States, emphasizing policy-related aspects such as perceived coverage quality, experiences with health access costs, access to a primary care physician, and financial literacy. This analysis explores the informational, social, and economic aspects influencing participation with preventive care, within the framework of the Affordable Care Act (ACA) and the ongoing underutilization of no-cost preventive services.

### 2.1 Perceived coverage quality

Perceived Coverage Quality in health insurance is a multidimensional construct encompassing Psychological Price, Health Insurance Satisfaction, and Ease of Claim Filing. Psychological Price refers to the subjective appraisal of premium affordability, reflecting the insured’s perception of the value received relative to cost [8], [40]. Health Insurance Satisfaction captures overall contentment with an insurance plan, including the breadth of coverage, provider networks, and customer service [41], [42]. Ease of Claim Filing addresses the perceived simplicity and efficiency of the claims process for both patients and providers [1], [43]. Together, these dimensions shape individuals’ global assessment of their insurance coverage and influence their confidence in navigating the healthcare system.

Perceived Coverage Quality is theorized to be closely associated with preventive health service utilization. Individuals who perceive their insurance as high-quality and affordable are more likely to pursue preventive care without fear of unexpected costs. High satisfaction with insurance can foster trust in the healthcare system and increase willingness to engage in recommended preventive measures. Additionally, streamlined claims processes reduce administrative burdens, further facilitating access to care. However, the relationship may be complex; greater perceived generosity of coverage could, in some cases, reduce the urgency to seek preventive care if individuals feel protected against future health risks. Prior research consistently demonstrates that positive perceptions of insurance coverage are linked to higher healthcare utilization, underscoring the importance of beneficiary trust and system navigation in policy design [44], [45].

### 2.2 Health access cost experience

Health Access Cost Experience is conceptualized as a composite of Healthcare Payment Inability, Cost-Driven Care Avoidance, and Unexpected Bills. Healthcare Payment Inability reflects difficulties in paying medical bills, often resulting in medical debt [8], [46]. Cost-Driven Care Avoidance describes the postponement or avoidance of necessary medical care due to financial concerns [40], [43]. Unexpected Bills arise when patients incur charges from out-of-network providers, frequently in emergency situations or through ancillary services at in-network facilities [1], [32]. Collectively, these experiences capture both the financial strain and behavioral consequences associated with healthcare costs.

The relationship between Health Access Cost Experience and preventive service utilization is nuanced. Economic theory suggests that individuals who have faced financial barriers or cost shocks may be motivated to utilize no-cost preventive services to avoid future expenses [10], [42]. The ACA’s removal of cost-sharing for preventive care was designed to leverage this behavioral incentive. However, this study indicates that the direct association between cost experiences and preventive care use may be attenuated when accounting for other factors such as financial knowledge. Understanding how cost experiences interact with insurance design and individual capability is critical for developing effective, equity-focused policies to increase preventive care uptake and reduce long-term healthcare expenditures.

### 2.3 Primary care physician

Access to a primary care physician (PCP) is defined by an ongoing relationship with a healthcare professional who serves as the first point of contact for most medical needs and coordinates comprehensive patient care. Primary care encompasses a wide range of services, including prevention, health promotion, disease management, counseling, and education [47]. The presence of a PCP is strongly associated with increased utilization of preventive services for several reasons. PCPs ensure continuity of care, develop a deep understanding of patients’ health histories, and provide tailored preventive recommendations and interventions [48], [49]. They are also well-positioned to deliver preventive services during routine visits, such as immunizations, screenings, and health education [50], [51], [52].

The accessibility and trusted relationship fostered by primary care increase the likelihood that patients will pursue timely preventive services and address health concerns proactively. Empirical evidence demonstrates that individuals with a regular source of care are significantly more likely to receive recommended preventive services, including vaccinations, screenings, and chronic disease monitoring [53]. Thus, strengthening patient-provider continuity is a central policy lever for improving public health and reducing disparities in preventive care utilization.

### 2.4 Financial knowledge

Objective financial knowledge refers to an individual’s understanding of core financial concepts — such as compound interest, inflation, risk diversification, and insurance — typically measured through standardized assessments [54], [55], [56], [57]. Financial literacy is posited to be positively associated with preventive health service utilization, as financially knowledgeable individuals are better equipped to make informed health investment decisions and recognize the long-term economic benefits of prevention.

Research shows that higher financial literacy is linked to healthier behaviors, greater use of preventive services, and more effective management of health-related costs [58], [59]. These individuals are more likely to engage in regular check-ups and screenings, which can facilitate early detection and management of health risks, potentially reducing future healthcare expenditures [60]. Furthermore, financial literacy enhances individuals’ ability to navigate complex insurance systems, understand coverage details, and make prudent financial choices regarding health, ultimately improving health outcomes and reducing financial distress [9].

Collectively, these theoretical perspectives underscore the importance of considering informational, relational, and economic factors — beyond the mere removal of cost barriers — in shaping preventive health behaviors and informing targeted policy interventions.

## 3 Methodology

### 3.1 Data source and sample design

The data for this study is derived from the 2024 Financial Inclusion Survey conducted by the Center of Insurance Policy and Research (CIPR) of the National Association of Insurance Commissioners (NAIC). The survey was administered between February and March 2024 using an online platform (Qualtrics), gathering responses from 3,611 individuals across the United States. To ensure adequate representation of minority groups, the survey employed prestratification weights during data collection, resulting in minority oversampling [61], [62], [63]. This approach is supported by research demonstrating that oversampling can improve estimate accuracy for underrepresented populations and reduce potential bias.

Post-stratification weights were applied using the ANESRAKE algorithm to achieve national representativeness. This statistical method adjusts sample weights to align with known population totals, thereby correcting for sampling bias and enhancing the overall representativeness of the survey data [64]. The survey was made representative based on a set of ranked variables, including region, age, education level, race, income level, and gender, utilizing the most recent US Census Bureau data for 2024. The survey encompassed a broad range of topics related to financial inclusion, such as health insurance, life insurance, retirement planning, financial literacy, and risk perception. Furthermore, it collected comprehensive demographic information, enabling a thorough analysis of financial behaviors and attitudes across various population segments.

### 3.2 Main variables

#### 3.2.1 Dependent variables

The dependent variable in this study is the use of preventive health services, assessed using a binary response to the query: “In the past 12 months, did you receive preventive health services?” Yes or no. This variable indicates whether respondents participated in any preventative treatment during the year prior to the survey. The binary characteristic of this variable enables the application of logistic regression and structural equation modeling analysis to examine the links between many independent variables and the likelihood of utilizing preventive health care.

#### 3.2.2 Independent variables

##### Key independent variables

Perceived Coverage Quality is a principal independent variable in this study, comprising three elements: affordability of the monthly health insurance premium, overall satisfaction with the health insurance plan, and ease of submitting a claim, each assessed on a 5-point scale. In the weighted logistic regression, these components are combined into a straightforward summative index, providing a practical, easily interpretable metric by assigning equal weights to each item. In contrast, Structural Equation Modeling (SEM) employs a latent variable approach, treating Perceived Coverage Quality as an unobserved construct indirectly measured by the three observed indicators. This latent variable approach allows for the explicit modeling of measurement error, accounts for shared variance among indicators, and can lead to more precise estimations [65], [66]. Employing both methodologies facilitates a comparison of results, enhancing robustness and bridging practical implications with theoretical insights [67]. The SEM also explicitly addresses potential endogeneity between Perceived Coverage Quality and Preventive Health Usage through an endogeneity correction path, improving the validity of causal interpretations. Additionally, SEM allows modeling of covariance between latent constructs, capturing interdependencies such as the relationship between Perceived Coverage Quality and Health Access Cost Experience, thereby offering a comprehensive understanding of the underlying dynamics.

Health Access Cost Experience is another principal independent variable, consisting of three binary indicators (yes/no) related to healthcare cost burdens over the previous 12 months: deferral of necessary healthcare due to cost, inability to settle medical bills, and encountering unexpected medical expenses. The weighted logistic regression aggregates these binary responses into an average index, offering a clear, interpretable measure of overall healthcare affordability difficulties. SEM, conversely, conceptualizes Health Access Cost Experience as a latent variable, allowing these three binary indicators to collectively reflect an underlying unobserved construct. This latent variable treatment accounts for measurement error, variance differences among indicators, and their correlated nature. Furthermore, SEM explicitly models covariance between Health Access Cost Experience and other latent and observed variables, such as financial literacy, providing deeper insights into the interplay of factors affecting preventive health behaviors.

The third primary independent variable, presence of a primary care physician, is assessed with a binary measure (Yes/No), indicating whether respondents have an established relationship with a healthcare professional who serves as their primary medical contact. Given its binary and straightforward nature, this variable is incorporated directly into both logistic regression and SEM analyses as an observed predictor, providing clarity and interpretability.

Objective financial literacy, the fourth primary independent variable, is evaluated using six binary questions related to understanding compound interest, inflation, and investment risk. A summative index is calculated for logistic regression analysis, with scores ranging from 0 to 6, clearly reflecting financial literacy levels. Due to the binary nature of these indicators and their potential limitations for reliably constructing a latent variable, SEM employs the summative financial literacy index directly as an observed variable. This approach ensures analytical simplicity, interpretability, and stability in the SEM model without compromising the validity or reliability of the construct.

##### Control variables

The study includes many control variables to address demographic and socioeconomic aspects that may affect the utilization of preventive health services. This encompasses a binary variable for gender, distinct dummy variables for race/ethnicity (White, Black, Asian, and Latino), a metric for household income level, and the greatest educational attainment achieved. The control variables are incorporated in the weighted logistic regression and the Structural Equation Model (SEM) to mitigate potential confounding effects and delineate the links between the primary independent variables and the utilization of preventive health services. Table 1 shows the descriptive statistics for all the variables used in the models.

**Table 1:** Descriptive statistics for all variables used in the models.

Variable	N	Mean	Median	SD	Var	IQR	Min	Max	Skewness	Kurtosis
Preventive health usage	3611	0.557	1.000	0.497	0.247	1.000	0	1	−0.229	1.050
Primary physician	3611	1.820	2.000	0.381	0.146	0.000	1	2	−1.700	3.870
Health access cost experience	3611	0.249	0.000	0.343	0.118	0.333	0	1	1.070	2.760
Perceived coverage quality	2997	4.140	4.330	0.802	0.644	1.330	1	5	−0.918	3.570
Financial knowledge	3611	3.260	3.000	1.590	2.520	3.000	0	6	−0.252	2.070
Female	3611	0.514	1.000	0.500	0.250	1.000	0	1	−0.055	1.000
White people	3611	0.576	1.000	0.494	0.244	1.000	0	1	−0.307	1.090
Black people	3611	0.132	0.000	0.339	0.115	0.000	0	1	2.170	5.720
Asian	3611	0.053	0.000	0.223	0.050	0.000	0	1	4.010	17.10
Latino	3611	0.182	0.000	0.386	0.149	0.000	0	1	1.650	3.720
Income	3611	4.880	5.000	2.100	4.390	4.000	1	9	−0.504	2.140
Education	3611	4.700	4.000	1.620	2.630	3.000	1	9	0.432	2.490

### 3.3 Data analysis

**Weighted Logistic Regression and Structural Equation Model (SEM)**

**Stage 1: Weighted Logistic Regression** The first stage estimates the logistic regression model for predicting preventive health usage, considering the survey weights:

$$\log\left(\frac{P\left(\text{Preventive Health Usage}=1|X\right)}{1-P\left(\text{Preventive Health Usage}=1|X\right)}\right)=\beta_{0}+\overset{p}{\underset{i=1}{\sum }}\beta_{i}X_{i}+\epsilon$$

Where $X_{i}$ represents the predictors such as demographic variables, health access cost (e.g., unexpected bills, cost-driven care avoidance, etc.), and Perceived Coverage Quality (e.g., Psychological price, Health insurance satisfaction, etc.). The predicted probabilities $\overset{ˆ}{P}\left(\text{Preventive Health Usage}\right)$ from this logistic model are used as the observed variable in the SEM.

**Stage 2: Weighted Structural Equation Model (SEM)**

*Measurement models:*

$$\eta_{1}=\lambda_{11}X_{1}+\lambda_{12}X_{2}+\lambda_{13}X_{3}\;+\;\alpha \;\eta_{3}\;+\;{ɛ}_{1},$$

$$\eta_{2}=\lambda_{21}Z_{1}+\lambda_{22}Z_{2}+\lambda_{23}Z_{3}\;+\;\pi \;FK\;+\;{ɛ}_{2},$$

where

• $\eta_{1}$ = Perceived Coverage Quality, measured by $X_{1},X_{2},X_{3}$,
• $\eta_{2}$ = Health Access Cost Experience, measured by $Z_{1},Z_{2},Z_{3}$,
• FK = Financial Knowledge (cross-construct predictor),
• α = endogeneity (feedback) coefficient from past usage $\eta_{3}$ into coverage,
• π = cross-construct coefficient from financial knowledge into cost experience,
• ${ɛ}_{1},{ɛ}_{2}$ = latent disturbance terms.

*Structural model:*

$$\eta_{3}\;=\;\beta_{1}\;\eta_{1}\;+\;\beta_{2}\;\eta_{2}\;+\;\underset{k}{\sum }\gamma_{k}\;W_{k}\;+\;{ɛ}_{3},$$

where

• $\eta_{3}$ = Preventive Health Usage,
• $W_{k}$ are the observed controls (income, education, primary physician, demographics),
• $\beta_{1},\beta_{2},\gamma_{k}$ are structural coefficients,
• ${ɛ}_{3}$ is the usage disturbance term.

*Latent covariance:*

$$\mathrm{Cov}\left({ɛ}_{1},{ɛ}_{2}\right)\;=\;\psi_{12},$$

acknowledging that unobserved factors driving coverage perceptions and cost experiences are themselves correlated.

*Incorporating sample weights:* The log-likelihood is weighted by $w_{i}$:

$$L\left(\Theta \right)=\overset{N}{\underset{i=1}{\sum }}w_{i}\;\ln P\left(\eta_{3i},\;X_{i},\;Z_{i},\;FK_{i}|\Theta \right),$$

where Θ={λ,α,π,β,γ,ψ} collects all measurement, structural, feedback and covariance parameters.

**Adjusted Estimation:** The weighted SEM uses weighted maximum likelihood estimation (W-MLE) to incorporate weights ($w_{i}$) during model estimation. This ensures that the latent constructs and structural relationships reflect the population characteristics as represented by the weighted sample.

#### Justification for analytical strategy

Structural Equation Modeling (SEM) complements weighted logistic regression by enabling a comprehensive analysis of complex relationships among observed and latent variables while accounting for measurement error. While weighted logistic regression provides robust estimates for binary outcomes by incorporating survey weights, SEM captures indirect effects, cross-construct linkages, and the interdependencies between multiple latent constructs (e.g., Perceived Coverage Quality, Health Access Cost Experience) influencing preventive healthcare access. SEM also explicitly addresses endogeneity by incorporating reciprocal pathways (feedback loops) between latent constructs, thus correcting for potential biases arising from reverse causation. Additionally, SEM includes cross-construct linkages, such as the influence of financial literacy on Health Access Cost Experience, which enriches the theoretical model and improves explanatory power. By modeling these latent variables and their relationships simultaneously, SEM offers a deeper understanding of the underlying processes that logistic regression alone might not fully capture [63], [68], [69]. This integrated approach strengthens the findings by explicitly modeling both direct and indirect pathways, accounting for endogeneity, reducing model misspecification, and enhancing the validity of causal inferences [68], [69], [70], [71].

## 4 Result

A series of weighted, design-based t-tests (Table 2) provides an essential first look at how preventive-care usage varies across key predictors in the survey sample. Individuals with a regular primary physician report mean preventive-care usage nearly 0.45 points higher than those without (t=−17.05, $p<0.001^{***}$), and those experiencing financial barriers such as unexpected bills, care avoidance, or payment difficulty show usage 0.21 points higher (t=5.29, $p<0.01^{**}$), suggesting that cost shocks may prompt greater engagement with covered preventive services. In contrast, gender (t=−0.69, p≥0.05) and certain race categories, including Asian and Latino status (t=−1.51, p≥0.05; t=−1.43, p≥0.05), do not exhibit significant mean differences, while White and Black respondents show moderate differences (t=4.64, $p<0.001^{***}$; t=−2.87, $p<0.01^{**}$). Significant differences are also observed for dichotomized continuous covariates such as perceived coverage quality (Δ=0.16, t=5.64, $p<0.001^{***}$), objective financial knowledge (Δ=0.14, t=5.46, $p<0.001^{***}$), income (Δ=0.22, t=9.17, $p<0.001^{***}$), and education (Δ=0.22, t=8.12, $p<0.001^{***}$). Employing t-tests as an initial step is particularly valuable, as they provide clear and interpretable evidence of associations between predictors and preventive-care usage, confirm adequate variability for further analysis, and guide variable selection for more sophisticated modeling. This diagnostic approach establishes a strong empirical foundation and effect-size intuition before advancing to multivariable weighted logistic regression and structural equation modeling (SEM), where more complex relationships, confounding, and latent constructs can be thoroughly examined in the context of health policy.

**Table 2:** Design-based t-tests of Preventive Health Usage by covariate.

Variable	t-value	Mean diff. ($\Delta \overset{¯}{y}$)	Signif.
*Binary covariates*			
Primary physician	−17.047	–0.450	***
Health access cost experience	5.289	0.211	***
Female	−0.690	–0.018	
White people	4.640	0.124	***
Black people	−2.874	–0.105	**
Asian	−1.512	–0.089	
Latino	−1.430	–0.048	
Perceived Coverage Quality	5.641	0.164	***
Financial Knowledge	5.460	0.144	***
Income	9.166	0.222	***
Education	8.117	0.216	***

*Notes:* *** p<0.001, ** p<0.01, * p<0.05, no star p≥0.05.

The weighted logistic regression model (Table 3) indicates that having a primary physician, facing increased health access cost challenges, perceiving enhanced coverage quality, and possessing superior financial knowledge all demonstrate statistically significant positive correlations with the utilization of preventive health services. Significantly, these fundamental variables — especially a consistent source of care (primary physician: Estimate = 1.86, p<0.001) and cost-related factors (Health Access Cost Experience: Estimate = 1.28, p<0.001) — exhibit some of the most substantial effect sizes, underscoring their pivotal role in the utilization of preventive services. Although income and education demonstrate notable positive effects, common demographic factors such as race and gender do not attain statistical significance in this model, indicating that, when controlling for coverage, cost, and financial literacy, these demographic variables may have a diminished direct influence on the utilization of preventive care.

**Table 3:** Regression Coefficients for Weighted Logistic Model Using svyglm.

Variable	Estimate	Std. Error	t Value	Pr(>|t|)	Signif.
Intercept	−5.48012	0.76644	−7.150	1.09e−12	***
Main variable					
Primary Physician (Yes)	1.85739	0.22874	8.120	6.75e−16	***
Health Access Cost Experience	1.27995	0.22390	5.717	1.19e−08	***
Perceived Coverage Quality	0.44927	0.09540	4.709	2.60e−06	***
Financial Knowledge	0.15367	0.04468	3.439	0.000591	***
Control variable					
Female	0.04234	0.13731	0.308	0.757865	
White people	0.35088	0.47773	0.734	0.462726	
Black people	0.23026	0.49306	0.467	0.640528	
Asian	−0.32499	0.59372	−0.547	0.584163	
Latino	0.20204	0.48172	0.419	0.674946	
Income	0.10689	0.04011	2.665	0.007736	**
Education	0.18652	0.05625	3.316	0.000925	***

*Notes:* *** p<0.001, ** p<0.01, * p<0.05, no star p≥0.05.

**Fig. 1:**
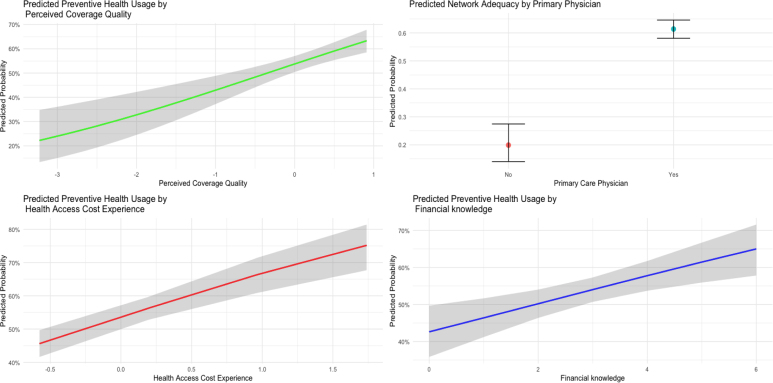
Predicted probabilities 2024 Financial Inclusion Survey .

Source: Author’s analysis.

Fig. 1 substantiates the findings from the weighted logistic regression by demonstrating distinct positive gradients in the projected probability of preventive health utilization across different levels of significant explanatory factors. In the upper-left panel, enhancing perceived coverage quality from its minimum to maximum observed values raises the expected preventive health utilization from around 10% to about 25%. The upper-right panel similarly contrasts persons lacking a primary care physician, with expected usage around 20%, to those possessing a primary care physician, whose chance nears 40%. A similar trend is shown in the lower-left panel, where an increase in health access cost experience from the lowest to maximal levels correlates with a rise in expected utilization from under 10% to almost 30%. As illustrated in the lower-right panel, augmenting financial knowledge from the lower spectrum of its distribution to the upper range increases anticipated preventive health utilization from roughly 15% to over 35%. These graphical data corroborate the regression findings by illustrating that enhancements in insurance-related views, healthcare provider connections, and financial comprehension correlate with significant and substantial increases in preventive health involvement.

While the weighted logistic regression in Table 3 showed that having a primary physician, experiencing greater health access cost barriers, perceiving higher coverage quality, and possessing stronger financial knowledge significantly increased the odds of preventive-care use, that method assumes each predictor is measured without error and posits strictly unidirectional effects.

**Fig. 3:**
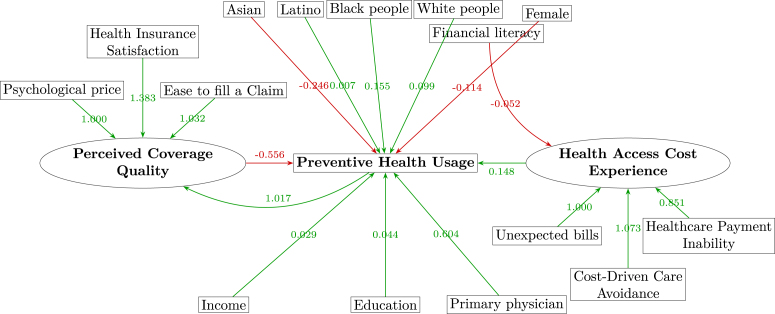
SEM diagram 2024 Financial Inclusion Survey.

In contrast, the Structural Equation Model (SEM) depicted in Fig. 3 represents *Perceived Coverage Quality* and *Health Access Cost Experience* as *latent constructs*, each measured by multiple indicators. Specifically, *Perceived Coverage Quality* is indicated by Psychological price, Health Insurance Satisfaction, and Easiness to Fill a Claim, while *Health Access Cost Experience* is represented by Healthcare Payment Inability, Cost-Driven Care Avoidance, and Unexpected Bills. This explicit modeling of latent variables accounts for measurement error inherent in insurance perceptions and cost-related experiences. Additionally, the SEM approach addresses potential endogeneity by modeling reciprocal relationships explicitly, particularly via a direct path from Preventive Health Usage back to *Perceived Coverage Quality*, thus correcting biases from possible reverse causation.

Author’s analysis.

Table 4 presents robust parameter estimates from the weighted SEM. All factor loadings are highly significant and substantively large (e.g., Health Insurance Satisfaction → Perceived Coverage Quality: λ=1.383, z=12.388, p<0.001; Cost-Driven Care Avoidance → Health Access Cost Experience: λ=1.073, z=11.984, p<0.001), confirming reliable measurement of the latent dimensions.

**Table 4:** Parameter estimates for the SEM model.

Parameter	Estimate	Std. Error	z-value	p-value	Signif.
**Latent Variables (Factor Loadings)**					

*Perceived Coverage Quality*					
Psychological price	1.000	–	–	–	–
Health Insurance Satisfaction	1.383	0.112	12.388	0.000	***
Easiness to fill a claim	1.032	0.097	10.645	0.000	***
*Health Access Cost Experience*					
Healthcare Payment Inability	1.000	–	–	–	–
Cost-Driven Care Avoidance	1.073	0.090	11.984	0.000	***
Unexpected bills	0.851	0.083	10.222	0.000	***

**DV: Preventive Health Usage**					

Perceived Coverage Quality	−0.556	0.171	−3.255	0.001	**
Health Access Cost Experience	0.148	0.112	1.320	0.187	
Primary Physician	0.604	0.099	6.078	0.000	***
Financial Knowledge	0.054	0.015	3.597	0.000	***
Female	−0.114	0.054	−2.115	0.034	*
White people	0.099	0.101	0.971	0.331	
Black people	0.155	0.116	1.330	0.184	
Asian	−0.246	0.134	−1.829	0.067	.
Latino	0.007	0.103	0.064	0.949	
Income	0.029	0.014	2.116	0.034	*
Education	0.044	0.016	2.795	0.005	**

**Endogeneity Correction Paths**					

Perceived Coverage Quality ∼ Preventive Health Usage	1.017	0.202	5.037	0.000	***

**Cross-Construct Linkages**					

Health Access Cost Experience ∼ Financial Knowledge	−0.052	0.009	−6.021	0.000	***

**Covariance**					

Perceived Coverage Quality ∼ Health Access Cost Experience	−0.069	0.013	−5.315	0.000	***

**Variances**					

Psychological price	0.766	0.061	12.582	0.000	***
Health Insurance Satisfaction	0.177	0.047	3.757	0.000	***
Easiness to fill a claim	0.646	0.068	9.491	0.000	***
Healthcare Payment Inability	0.637	0.068	9.430	0.000	***
Cost-Driven Care Avoidance	0.513	0.057	8.957	0.000	***
Unexpected bills	0.672	0.045	15.063	0.000	***
Preventive Health Usage	0.325	0.072	4.509	0.000	***
Perceived Coverage Quality (latent)	0.472	0.098	4.828	0.000	***
Health Access Cost Experience (latent)	0.090	0.011	7.933	0.000	***

The structural model reveals a significant negative direct effect of *Perceived Coverage Quality* on Preventive Health Usage (β=−0.556, z=−3.255, p=0.001), suggesting paradoxically that when individuals perceive their insurance coverage as highly generous, they may feel less urgency to utilize preventive health services. Conversely, the direct path from *Health Access Cost Experience* to Preventive Health Usage becomes non-significant (β=0.148, z=1.320, p=0.187) after controlling for financial knowledge. This finding implies that enhanced financial literacy reduces perceived cost barriers, mitigating their direct influence on preventive care usage. Consistent with logistic regression results, having a primary physician (β=0.604, z=6.078, p<0.001) and financial knowledge (β=0.054, z=3.597, p<0.001) remain positively and significantly associated with preventive health utilization. Among demographic variables, income (β=0.029, z=2.116, p=0.034) and education (β=0.044, z=2.795, p=0.005) maintain significant positive associations, whereas gender and race/ethnicity do not reach significance.

**Fig. 2:**
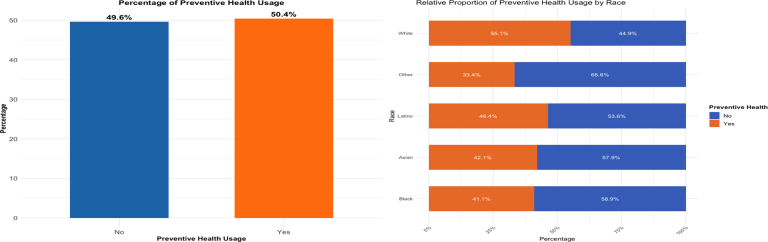
Relative proportion: preventive health usage among the insured in the US 2024 Financial Inclusion Survey .

An essential feature of this SEM is the inclusion of an endogeneity correction path from *Preventive Health Usage* back to *Perceived Coverage Quality* (β=1.017, z=5.037, p<0.001), explicitly capturing how preventive care use positively shapes subsequent coverage perceptions. Additionally, the cross-construct path from *Financial Knowledge* to *Health Access Cost Experience* (β=−0.052, z=−6.021, p<0.001) indicates that improved financial literacy reduces perceived financial barriers. The significant negative covariance between *Perceived Coverage Quality* and *Health Access Cost Experience* (ψ=−0.069, z=−5.315, p<0.001) further reveals that higher perceptions of coverage quality coincide with fewer cost-related obstacles, possibly reducing individuals’ urgency to seek preventive care—thus partly explaining persistently low usage rates even among the insured (Fig. 2).

Author’s analysis.

Indeed, Fig. 2 confirms inadequate utilization of preventive health services among insured respondents, demonstrating persistent barriers despite coverage. Particularly notable are racial disparities, with Black (58.9% non-use) and Asian individuals (57.9% non-use) exhibiting lower usage compared to White respondents (55.1% usage). These findings align with existing health economics literature attributing such differences to socioeconomic status, systemic inequities, and cultural barriers [8], [40]. Compared to logistic regression relying on simplistic indices, the SEM approach offers a robust and theoretically grounded framework that explicitly quantifies measurement error, reciprocal causation, and complex relationships among latent constructs, providing a comprehensive explanation of preventive healthcare behaviors.

Fig. 3 visually complements the numeric insights from Table 4, explicitly highlighting the structural relationships among latent constructs and observed variables within the SEM. The diagram underscores the central position of *Health Access Cost Experience* and *Perceived Coverage Quality* as mediators linking demographic and socioeconomic factors to preventive health usage. Additionally, it clarifies reciprocal relationships, such as prior preventive service utilization enhancing subsequent coverage perceptions, and illustrates how financial literacy can mitigate perceived financial barriers. The reversed (negative) paths observed from demographic variables (e.g., Asian) to preventive health usage reflect statistical artifacts rather than theoretically meaningful relationships. These artifacts arise when modification indices suggest adding empirical paths to address residual correlations unexplained by the model’s current structure. Importantly, these artifacts do not undermine the conceptual validity or overall integrity of the model, particularly given the strong theoretical grounding of the primary constructs and their pathways. The following section further reinforces this conclusion by providing a comprehensive model fit assessment, demonstrating the SEM’s robustness and appropriateness.

### Model fit assessment

**Fig. 4:**
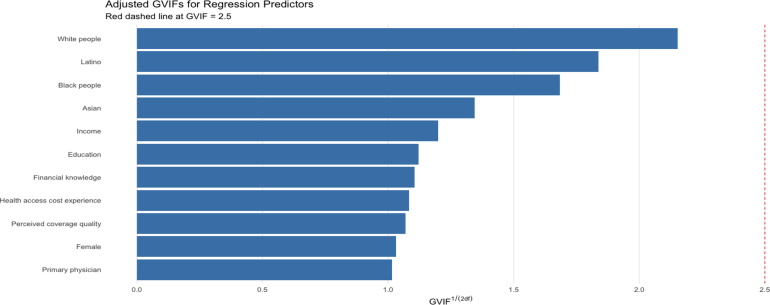
Adjusted GVIFs for Regression Predictors 2024 Financial Inclusion Survey .

Table 5 reports several standard fit indices. The Comparative Fit Index (CFI = 0.916) and Tucker–Lewis Index (TLI = 0.890) both exceed 0.80 — and CFI even surpasses the conventional 0.90 threshold — indicating substantial improvement over the null model [72], [73], [74]. The Root Mean Square Error of Approximation (RMSEA) of 0.049 (90% CI [0.044,0.054] ) falls below the 0.05 “close-fit” benchmark, suggesting minimal per-degree-of-freedom approximation error [75], [76]. Finally, the Standardized Root Mean Square Residual (SRMR) of 0.036 is well under the 0.08 cutoff [74], indicating very small average standardized residuals. Taken together — especially given the RMSEA and SRMR — the indices show that the model reproduces the observed data exceptionally well, justifying substantive interpretation of its parameter estimates. Fig. 4 presents the adjusted variance inflation factors (adjusted for degrees of freedom) of the variables used in the model, confirming that multicollinearity is not a concern, as all values are substantially below the conventional threshold of 5.

**Table 5:** Key fit indices for the SEM specification.

Fit Index	Value
Comparative Fit Index (CFI)	0.916
Tucker–Lewis Index (TLI)	0.890
Root Mean Square Error of Approx. (RMSEA)	0.049
RMSEA 90% CI lower	0.044
RMSEA 90% CI upper	0.054
Standardized RMS Residual (SRMR)	0.036

Author’s analysis.

## 5 Discussion

The present study illuminates the intricate and layered nature of preventive health service utilization among insured adults in the United States, drawing upon both weighted logistic regression and Structural Equation Modeling (SEM) to unravel the underlying mechanisms. These findings reinforce evidence from public health and health economics that consistently demonstrate how structural, informational, and relational factors — far beyond financial barriers alone — drive engagement with preventive care [77], [78], [79], [80], [81]. By harnessing nationally representative data and leveraging advanced statistical approaches, this research sheds light on critical policy-relevant levers that can help close persistent gaps in the uptake of no-cost preventive services, which continue despite the major access expansions ushered in by the Affordable Care Act (ACA) [2].

A central contribution of the analysis is its demonstration that access to a primary care physician and robust financial knowledge are the strongest, most consistent predictors of preventive service use, even after controlling for income, education, and demographic variables. This finding confirms and extends prior studies that highlight the primacy of patient-provider continuity and health literacy in promoting proactive health behaviors [7], [27], [50]. SEM results reinforce that these factors exert both direct and indirect effects: they not only foster trust and information-sharing, but also empower individuals to better navigate cost complexities and access the full benefits of insurance coverage. The existence of highly significant and positive effects for both variables supports targeted policy recommendations, such as incentivizing continuous primary care enrollment, integrating health and financial education into insurance onboarding, and supporting outreach programs aimed at low-literacy populations.

The nuanced relationship between perceived coverage quality and preventive use revealed by SEM highlights an important paradox. While one might expect that satisfaction and confidence in insurance would universally promote greater use, the analysis shows that very generous or high-quality perceived insurance coverage can at times dampen the urgency to seek preventive care. This phenomenon, consistent with theories from behavioral economics and risk compensation, suggests the existence of a “complacency effect”, where perceived protection reduces perceived need for preventive action [82], [83]. Accordingly, policy should not only guarantee comprehensive coverage but should also actively prompt and facilitate timely use—through features like reminders, automatic scheduling, and default primary care assignments, all of which are shown to be effective in shifting preventive health behaviors.

The interplay of financial literacy and health access cost experience offers additional insight into how informational and experiential factors mediate behavioral responses to the health system. While cost barriers have traditionally been seen as straightforward deterrents to care, the current findings reveal a more dynamic reality. The direct effect of negative cost experiences on preventive care use is explained or mitigated in the presence of strong financial knowledge, supporting multidimensional interventions that combine financial education with improvements to insurance design [84], [85]. This also underscores the importance of recognizing both real and perceived barriers: individuals with greater financial capability are better equipped to interpret and respond to insurance communications, manage medical bills, and make informed use of covered services, reducing avoidance and delaying less care for financial reasons [85].

Importantly, the relative insignificance of race and gender in multivariable models — once structural and informational mediators are entered — signals a shift in the underlying sources of observed disparities in preventive uptake. This supports a new emphasis on addressing root causes by targeting resource, knowledge, and provider barriers, rather than defaulting to surface-level demographic targeting. These findings have resonance for addressing equity and advancing population health, since historical disparities in access and outcomes are likely to persist if policymakers do not address trust, navigation, and infrastructure gaps in tandem with insurance expansion [6], [8].

Another notable contribution of the SEM approach is its ability to explicitly model feedback loops and address endogeneity, shedding light on the bidirectional nature of engagement and perception. The finding that utilization of preventive health services can itself enhance perceptions of insurance coverage quality points to a virtuous cycle: participation breeds confidence, which in turn can fuel further engagement. This empirically supports the case for “first touch” or low-threshold interventions that activate entry into preventive care, as the gains may multiply beyond a single encounter [86].

These results are also highly salient in the context of contemporary policy debates and legal challenges to the scope of preventive coverage. The unacceptably low overall preventive utilization rates — even in a no-cost landscape — demonstrate that resolving coverage gaps does not resolve informational, psychological, and relational barriers. Persistent disparities by race and other sociodemographic traits, unraveling when structural variables are modeled, confirm that public health efforts must embrace multifaceted, targeted, and equity-driven reforms [36], [37].

Finally, this study expands the evidence base by demonstrating the limits of insurance expansion as a singular strategy and clarifies the importance of informational and relational policy levers in shifting preventive health behaviors. Policies that cultivate trust in coverage, enhance system navigation, promote ongoing relationships with primary care, and boost health and financial literacy are essential for raising preventive service utilization, closing gaps, and ultimately reducing long-term health inequities in the United States.

## 6 Conclusion

This study underscores that the uptake of preventive health services among insured individuals in the United States is shaped by a multifaceted combination of informational, relational, and experiential factors—not simply by the removal of direct financial barriers. While the Affordable Care Act (ACA) has significantly expanded access by eliminating cost-sharing for preventive services, our findings reveal that perceived coverage quality, continuous access to a primary care physician, and financial literacy all exert independent and substantial influences on preventive care participation [69], [87], [88]. Notably, once these factors are modeled, traditional demographic variables such as race and gender are no longer significant predictors, emphasizing the need for policies that move beyond demographic targeting and address underlying systemic and educational determinants.

A key strength of this research is the application of weighted Structural Equation Modeling (SEM), which enables the explicit modeling of latent constructs, the correction for endogeneity, and the investigation of complex, reciprocal pathways between perceptions, experiences, and behaviors. These methodological advances provide a more comprehensive and nuanced understanding of the many drivers of preventive care utilization.

The policy implications are clear: efforts to increase preventive health engagement should expand beyond broadening insurance coverage alone. Interventions should foster trust in insurance, enhance transparency and understanding of benefits, reinforce the patient-provider relationship through primary care continuity, and improve financial literacy within healthcare settings. By addressing these structural and informational barriers, policymakers and healthcare leaders can design more equitable, targeted, and effective strategies to advance public health goals and fulfill the ACA’s promise of expanded, meaningful access to preventive care.

## 7 Limitation

This study’s findings should be interpreted in light of several important limitations. The reliance on self-reported, cross-sectional survey data raises concerns about recall error and social desirability bias, as participants may not accurately recount their use of preventive services or might exaggerate their engagement to align with perceived social expectations. The cross-sectional design further restricts the analysis to a single point in time, meaning that any observed associations between financial literacy, primary care access, insurance perceptions, and preventive care utilization cannot be definitively interpreted as causal relationships—even though advanced modeling such as SEM was employed to help address endogeneity and reciprocal effects. Unmeasured factors, including cultural beliefs, political attitudes, provider availability, and local health system features, were not fully captured by the survey instrument and may confound the reported associations. Moreover, measurement error is possible in the construction of latent variables, and sampling bias or rapidly shifting healthcare policies could limit the broader applicability of the result [21].

## 8 Future research

To advance the evidence base and address these limitations, future research should explicitly utilize longitudinal or quasi-experimental study designs, such as panel studies or natural experiments, to clarify causal directions and capture how changes in financial literacy, insurance coverage, and primary care relationships shape preventive health behaviors over time. These approaches would allow researchers to observe the dynamic interplay among policy, socio-economic environments, and individual experiences as they relate to prevention. Additionally, future work should move beyond simple measures of utilization and focus on evaluating the clinical quality and appropriateness of preventive services to ensure that increased uptake confers genuine health benefits. A deeper investigation of sociopolitical factors — such as trust in government and perceptions of policy fairness — is also warranted, as these aspects can strongly influence engagement in preventive systems. Targeted efforts to understand and intervene on structural, ideological, and equity barriers will be key for developing more effective and inclusive public health strategies.

## Ethical committee approval

Not applicable, as the research did not involve human participants, animals, or data requiring oversight by an ethics committee.

## Funding statement

This research did not receive any specific grant from funding agencies in the public, commercial, or not-for-profit sectors. The project was self-funded, underscoring my commitment to advancing the field through independent efforts.

## Declaration of competing interest

The authors declare that they have no known competing financial interests or personal relationships that could have appeared to influence the work reported in this paper.
